# All Is Swell: A Case Report of Insulin Edema Syndrome

**DOI:** 10.7759/cureus.112221

**Published:** 2026-07-07

**Authors:** Adelaine Espiritu, Nishtha Nigam, Patricia Perez de Tagle, Sankalp Acharya, Axle Untalan, Suryansh Atreya, Rutuja Challawar, Patricia Salonga, Peter N Fish

**Affiliations:** 1 Internal Medicine, Monmouth Medical Center, Long Branch, USA

**Keywords:** adverse drug reaction, generalized anasarca, insulin-induced edema, insulin-naive, newly diagnosed diabetes mellitus

## Abstract

Insulin edema syndrome is a rare and underrecognized complication of insulin initiation or intensification, characterized by peripheral or generalized edema. Its pathophysiology is multifactorial, involving renal salt retention, increased capillary permeability, and vasodilation, and it is often a diagnosis of exclusion.

We report the case of a 41-year-old man with newly diagnosed type 2 diabetes mellitus presenting with hyperglycemia (772 mg/dL) and marked weight loss. After initiation of insulin glargine and lispro, he developed rapid-onset generalized edema, including scrotal and lower extremity swelling, weight gain of 32 pounds, dyspnea, and pleural effusions. An extensive workup excluded cardiac, renal, hepatic, infectious, autoimmune, and protein-losing enteropathy etiologies. The edema worsened with higher insulin doses despite treatment with diuretics and corticosteroids. Transition to NPH (neutral protamine Hagedorn) and regular insulin, with adjunct dapagliflozin, resulted in rapid resolution of the edema, allowing discontinuation of the diuretics.

Risk factors for insulin edema include newly diagnosed diabetes, rapid glycemic correction, low body weight, and high insulin doses. While generally self-limiting, severe or refractory cases may require diuretics or modification of the insulin regimen. Recognition is critical to avoid unnecessary interventions and to manage symptoms effectively.

Insulin edema syndrome should be considered in patients presenting with unexplained edema after insulin initiation. Dose adjustment or switching insulin analogs, combined with supportive management, can lead to rapid improvement. Awareness of this condition may help prevent misdiagnosis and unnecessary invasive procedures.

## Introduction

Insulin edema syndrome (IES) is a rare and likely underrecognized complication that occurs following the initiation or rapid intensification of insulin therapy, particularly in patients with newly diagnosed or poorly controlled diabetes mellitus [1-3]. Although first described decades ago, IES remains infrequently reported and may be overlooked because of its self-limited course and nonspecific presentation [2,4]. Patients typically present with acute-onset peripheral edema, which can progress to generalized edema or anasarca, often accompanied by rapid weight gain, raising concern for cardiac, renal, or hepatic pathology [2-4].

The pathophysiology of IES is not fully understood but is thought to be multifactorial. Proposed mechanisms include insulin-induced renal sodium retention (antinatriuretic effect), transient hyperaldosteronism, and inappropriate antidiuretic hormone secretion [2,4]. Additionally, insulin may increase capillary permeability and contribute to fluid extravasation, particularly during rapid glycemic correction [3,4]. These processes may be exacerbated by osmotic shifts associated with sudden improvements in chronic hyperglycemia.

IES is a diagnosis of exclusion after more common causes of edema, including heart failure, nephrotic syndrome, and liver disease, have been ruled out [2,4]. Recognition of this entity is important to avoid unnecessary diagnostic evaluation and inappropriate treatment escalation. Management is generally supportive, consisting of cautious insulin dose adjustment, dietary sodium restriction, and short-term use of diuretics when clinically indicated, with most cases resolving spontaneously within weeks [2,4].

## Case presentation

A 41-year-old man with no significant past medical history other than being severely underweight (BMI 13.7 kg/m²) presented for evaluation after a mechanical fall. He was incidentally found to be hyperglycemic, with a glucose level of 772 mg/dL on admission. Other laboratory tests revealed borderline elevated beta-hydroxybutyrate (2.1 mmol/L), venous pH of 7.44, creatinine of 1.33 mg/dL, transaminitis (alanine aminotransferase (ALT) 183 U/L, aspartate aminotransferase (AST) 174 U/L, alkaline phosphatase (ALP) 188 U/L), and HbA1c >14% (Table 1). Dedicated imaging studies, including right upper quadrant ultrasound and CT of the abdomen and pelvis, were performed to evaluate these abnormal findings; however, they did not reveal any acute or remarkable findings. 

**Table 1:** Pertinent laboratory findings on index admission and readmission

Laboratory Test	Patient Value	Reference Range
Beta-hydroxybutyrate	2.1 mg/dL	<4.16 mg/dL
Venous pH	7.44	7.35-7.45
Serum creatinine	1.33 mg/dL	0.6-1.2 mg/dL
Alanine transaminase (ALT)	183 U/L	10-43 U/L
Aspartate aminotransferase (AST)	174 U/L	13-41 U/L
Alkaline phosphatase (ALP)	188 U/L	41-119 U/L
Hemoglobin A1c (HbA1c)	>14%	<5.7 %
B-type natriuretic peptide (BNP)	582 pg/mL	≤100 pg/mL
Hemoglobin (Hb)	8.4 g/dL	13.5-18 g/dL
Serum sodium (Na)	146 mmol/L	135-145 mmol/L
Serum albumin	2.8 g/dL	3.5-5.0 g/dL
Serum glucose (readmission)	248 mg/dL	70-100 mg/dL
Urine microalbumin	893 mcg/mL	<30 mcg/mL
Urine creatinine	148.3 mg/dL	-
Urine microalbumin/creatinine ratio	602.2 mcg/mg	≤30 mcg/mg creat

Further history revealed that he had experienced polyphagia, polydipsia, and over 50 pounds of weight loss over the previous year, as well as weakness that likely led to his fall. His family history was significant for type 2 diabetes mellitus in his father and sister. He smoked approximately five mild cigars every two weeks for 20 years (equivalent to ~0.4 cigarette pack-years) but denied alcohol or illicit drug use and had not seen a physician in 20 years. He denied chest pain, dyspnea, abdominal symptoms, or lower extremity swelling.

The patient underwent further evaluation for diabetes mellitus in the setting of the above symptoms and marked hyperglycemia. Glutamic acid decarboxylase (GAD) antibody testing was negative, while the serum C-peptide level was low. Based on the overall clinical evaluation, the patient was diagnosed with presumed advanced, previously untreated type 2 diabetes mellitus with significant endogenous insulin deficiency. He was managed with sliding-scale insulin, which reduced his blood glucose from an initial 772 mg/dL to 115-283 mg/dL within one day. Along with this, his liver enzyme levels also improved slightly, and his creatinine decreased to a normal level of 0.91 mg/dL. The patient was subsequently discharged home on insulin glargine 10 units nightly and a lispro sliding scale.

**Figure 1:**
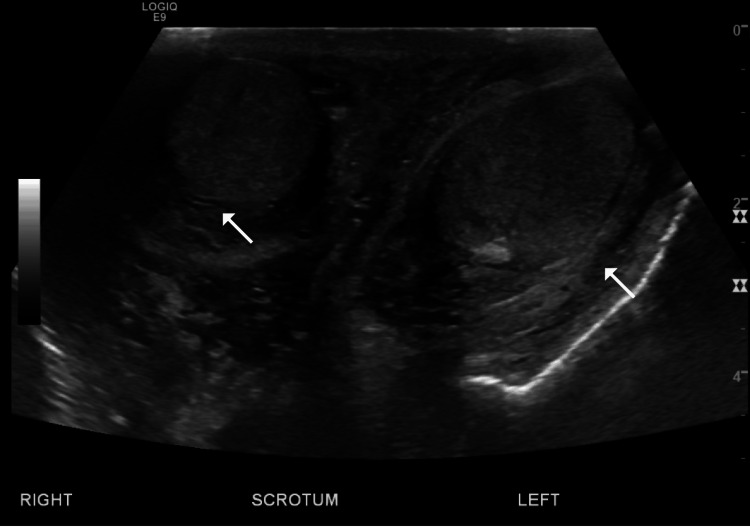
Bilateral scrotal edema Scrotal edema is characterized by an "onion skin" pattern on ultrasound, where the thickened scrotal wall is striated or layered in appearance, as indicated by arrows.

**Figure 2:**
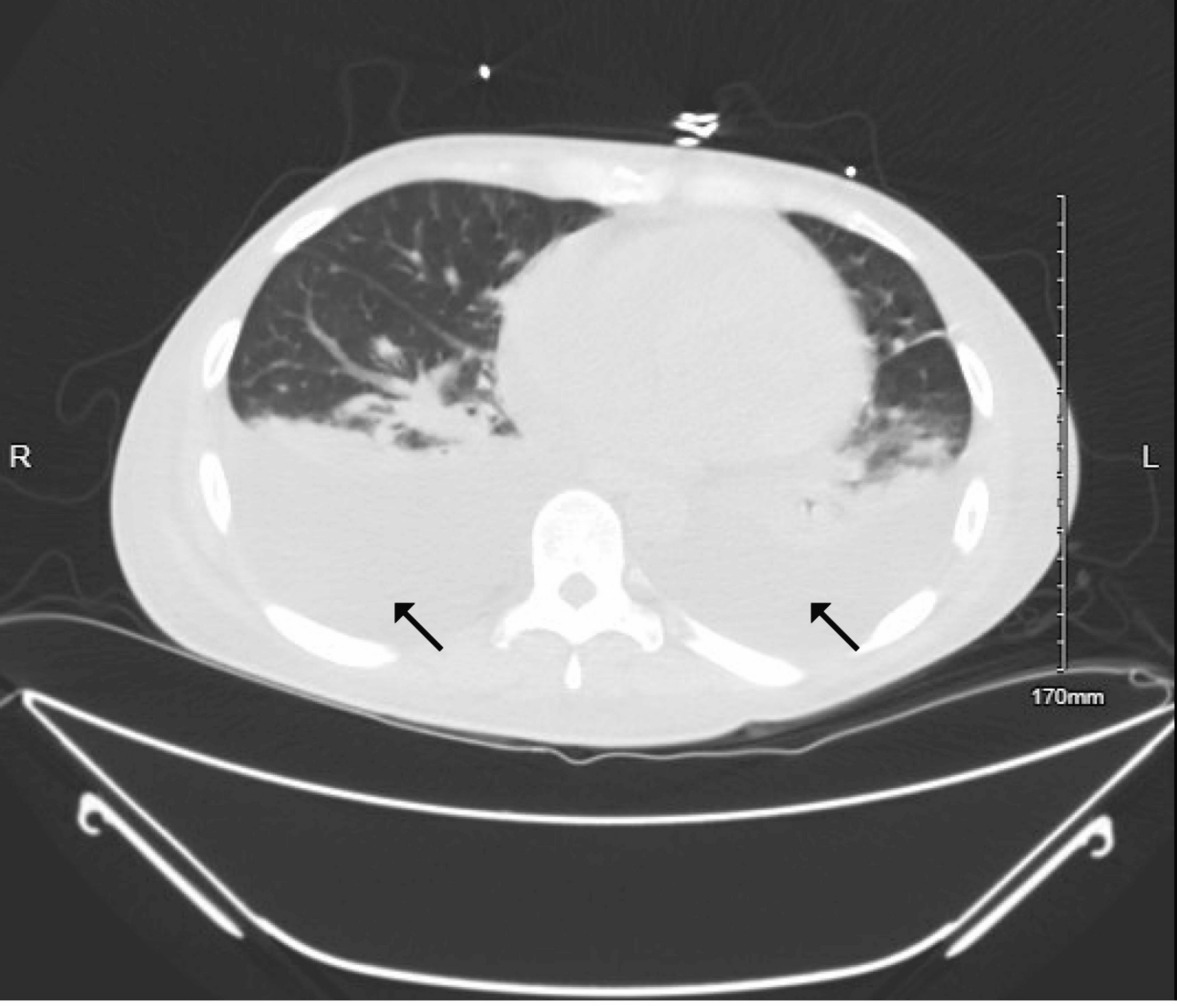
Large bilateral pleural effusions Large bilateral pleural effusions are indicated by the arrows on a non-contrast CT scan of the chest.

Within one week of discharge, the patient developed progressive lower extremity swelling, weight gain, and dyspnea on exertion, which did not improve with physical therapy. Ten days later, he returned to the hospital with scrotal and bilateral leg edema, a 32-pound weight gain, and hypoxemia requiring high-flow nasal cannula oxygen. Physical examination was significant for scrotal and penile swelling along with 2+ lower extremity edema, none of which had been present during his index admission. Laboratory evaluation was notable for an elevated B-type natriuretic peptide (BNP) level of 582 pg/mL, anemia (hemoglobin, 8.4 g/dL), hypernatremia (sodium, 146 mmol/L), hypoalbuminemia (2.8 g/dL), hyperglycemia (248 mg/dL), and elevated liver enzymes (AST, 66 U/L; ALT, 115 U/L; ALP, 116 U/L). Scrotal ultrasonography (Figure 1) demonstrated findings suggestive of epididymo-orchitis, and the patient was empirically treated with oral levofloxacin. However, urinalysis showed no evidence of urinary tract infection, and testing for gonorrhea and chlamydia was negative. Although a localized inflammatory process could not be definitively excluded, it was considered insufficient to account for the marked bilateral scrotal swelling in the setting of severe generalized anasarca, large bilateral hydroceles, bilateral pleural effusions, and ascites. Computed tomography angiography of the chest, abdomen, and pelvis demonstrated bilateral alveolar consolidations with large bilateral pleural effusions (Figure 2), severe anasarca, large bilateral hydroceles, and mild ascites, without evidence of hepatic or renal pathology.

Given the elevated BNP level and diffuse volume overload, a cardiac etiology was investigated. Transthoracic echocardiography demonstrated a preserved left ventricular ejection fraction of 60%-65%, normal global longitudinal strain (−18.3%), a small left ventricular cavity with moderate concentric hypertrophy, and grade I diastolic dysfunction. The left atrium was normal in size, the right ventricle was normal in size and function, and the inferior vena cava was normal in size with normal collapsibility. Mild-to-moderate tricuspid regurgitation was present, with an estimated pulmonary artery pressure of 35-40 mmHg. A small pericardial effusion was also noted. Cardiology was consulted and, based on the overall clinical and echocardiographic findings, considered a primary cardiac etiology unlikely to account for the severity of the patient's generalized edema. 

The patient underwent thoracentesis on hospital day 6, with pleural fluid studies consistent with a transudative effusion. An extensive infectious, autoimmune, and metabolic evaluation was unrevealing, including testing for HIV, mumps, coxsackievirus antibodies, Viral hepatitis, antineutrophil cytoplasmic antibodies (ANCA), anti-glomerular basement membrane (anti-GBM) antibodies, alpha-1 antitrypsin, serum free light chains, antinuclear antibodies (ANA), anti-smooth muscle antibodies, serum protein electrophoresis, and ceruloplasmin. Urine studies showed an albumin concentration of 893 µg/mL, a creatinine concentration of 148.3 mg/dL, and an albumin-to-creatinine ratio of 602.2 µg/mg. A 24-hour urine collection demonstrated protein excretion of 1,279.91 mg/day. Although the patient had moderate proteinuria, hypoalbuminemia (2.8 g/dL), and severe undernutrition (BMI, 13.7 kg/m²), he had no anasarca or other clinical evidence of generalized fluid accumulation on index admission, and renal function was preserved at that time. The degree of proteinuria was below the nephrotic range. Protein-losing enteropathy was considered in the differential diagnosis of hypoalbuminemia. The patient reported normal stool consistency and denied diarrhea, bloating, abdominal pain, or changes in bowel habits. Given the absence of gastrointestinal symptoms or other clinical features suggestive of an underlying gastrointestinal disorder, protein-losing enteropathy was considered less likely, and further objective testing was not pursued.

On hospital day 8, a repeat chest CT was obtained due to worsening oxygen requirements. It revealed multilobar pneumonia and the interval development of large bilateral pleural effusions, prompting repeat bilateral thoracenteses, which yielded 1.2 L of transudative fluid from each side. His antibiotic regimen was broadened from levofloxacin to cefepime, and intravenous steroids were initiated in the setting of elevated inflammatory markers (erythrocyte sedimentation rate (ESR) and C-reactive protein (CRP)). 

Alongside the evaluation of the patient’s acute anasarca, his persistent hyperglycemia was managed with progressive intensification of insulin therapy. Insulin glargine was increased to 35 units daily, and insulin lispro was increased to 10 units three times daily. Despite treatment with diuretics, corticosteroids, and intensification of the insulin regimen, his edema and oxygen requirements continued to worsen. On hospital day 9, given concern for insulin edema syndrome, the insulin regimen was changed from glargine and lispro to Humulin 70/30 and regular insulin, while dapagliflozin was initiated concurrently. On hospital day 15, he was successfully weaned off from high-flow nasal cannula to room air while maintaining adequate oxygen saturation. Scrotal edema and bilateral lower extremity swelling were documented to have improved over the course of the last five days before his discharge. His weight decreased from 79.9 kg on admission to 72.8 kg at discharge. Given the close temporal relationship between insulin initiation and the abrupt development of anasarca, together with the absence of a more compelling alternative etiology, insulin edema syndrome was considered the most likely diagnosis.

## Discussion

Insulin edema syndrome is a rare complication of the initiation or intensification of an insulin regimen and can occur in patients with either type 1 or type 2 diabetes mellitus. This syndrome presents as peripheral or generalized edema and is a diagnosis of exclusion. It can manifest as anasarca and effusions within days to weeks of insulin initiation or dose titration [3,5]. In more severe cases, it can lead to overt cardiac and renal failure symptoms. The risk of insulin edema syndrome increases in the following scenarios: newly diagnosed diabetes mellitus [1], poorly controlled or chronic hyperglycemia before insulin initiation or intensification [3], rapid improvement in glycemic control after starting or escalating insulin therapy rather than gradual titration [3,4,6], low body weight or underweight status and undernutrition [1,3,7], and high doses of insulin, especially in the context of insulin resistance or insensitivity [4]. Additional potential contributors, such as the presence of comorbidities (e.g., hepatic dysfunction affecting fluid homeostasis) and autonomic or sensory neuropathy associated with rapid glycemic shifts, have been proposed, although these factors are less well documented in the literature.

Mild to moderate insulin-induced edema occurs in 1%-17% of insulin-treated patients [4]. The first report of significant generalized edema dates to 1928 [4]. A case series spanning 1928-2004 described 31 cases (mean age, 33.8 years), with a higher prevalence in females (1:1.8), onset 2-30 days after insulin initiation, and weight gain of 1.8-20 kg [4].

The pathophysiology is likely multifactorial, as insulin promotes renal salt retention via Na+-K+ ATPase and Na+/H+ exchanger stimulation, causes vasodilation mediated by nitric oxide, and increases capillary permeability through vascular endothelial growth factor expression. Acute insulin elevations can also reduce serum albumin and increase urinary protein excretion, making renal salt retention and increased capillary permeability the principal mechanisms [4].

Its rarity is probably attributable to the need for a specific combination of predisposing circumstances, as outlined above, and to its generally self-limiting course with conservative management.

In retrospect, our patient had several potential risk factors for insulin edema syndrome, including severe undernutrition, markedly low BMI, prolonged uncontrolled diabetes, and significant endogenous insulin deficiency. In patients with similar clinical features, gradual initiation and cautious titration of insulin, avoidance of overly rapid glycemic correction when clinically feasible, and close monitoring for early weight gain, peripheral edema, and fluid accumulation may help identify this complication before progression to severe anasarca. Attention to baseline nutritional status, serum albumin, renal function, and fluid balance may also help identify patients who are particularly susceptible to fluid shifts. However, insulin edema syndrome remains rare and unpredictable, and no established evidence-based preventive strategy currently exists. Importantly, necessary insulin therapy should not be withheld in patients with severe hyperglycemia; rather, heightened clinical surveillance and individualized titration may allow earlier recognition and management.

In our patient, it is important to note that the addition of a sodium-glucose cotransporter 2 (SGLT2) inhibitor may have additionally promoted glycosuria and, in effect, osmotic diuresis and natriuresis. The simultaneous initiation of dapagliflozin and modification of the insulin regimen precludes attribution of the clinical improvement to either intervention alone and represents a confounding factor in interpreting the treatment response. Accordingly, the diagnosis of insulin edema syndrome was based primarily on the abrupt development of generalized edema following insulin initiation and intensification, together with the absence of a more compelling primary cardiac, hepatic, or renal etiology, rather than on the subsequent response to the change in insulin therapy.

Although generally self-limiting, management may require diuretics in severe or refractory cases [3,4]. Furosemide, spironolactone, epinephrine, or ephedrine have been used [4]. Dose reduction or switching insulin analogs has improved edema in reported cases [6,8].

## Conclusions

This report describes insulin edema syndrome, a rare and poorly understood complication of insulin therapy. As a diagnosis of exclusion, it requires careful evaluation for more common causes of generalized edema. In this case, cardiac, renal, hepatic, infectious, autoimmune, and metabolic etiologies were investigated through echocardiography, specialist assessment, urinary protein quantification, imaging, pleural fluid analysis, and extensive laboratory testing. Although severe undernutrition, hypoalbuminemia, and subnephrotic proteinuria likely increased susceptibility to edema, they were insufficient to fully explain its abrupt onset and severity. The close temporal relationship between insulin initiation and the rapid development of profound anasarca, in the absence of a more compelling alternative etiology, supported insulin edema syndrome as the most likely diagnosis.

Clinicians should recognize this rare complication because it can progress from peripheral edema to severe multisystem fluid accumulation and cardiopulmonary compromise. Although no established treatment strategy exists, management may include supportive care, judicious use of diuretics, and individualized adjustment of insulin therapy. Close monitoring of high-risk, insulin-naive patients after treatment initiation may facilitate early recognition and prevent progression to complications requiring invasive intervention.

Future studies should explore the development of risk stratification and monitoring protocols based on the number and combination of concurrent risk factors present at insulin initiation. Such protocols may help identify patients who would benefit from closer surveillance and more cautious insulin titration.
